# P-1131. Comparison of Vancomycin Use Before and After the Implementation of an Antimicrobial Stewardship Program in a Pediatric Reference Hospital in Mexico

**DOI:** 10.1093/ofid/ofae631.1318

**Published:** 2025-01-29

**Authors:** Lindsay A Concha-Mora, Gloria R Ayala Villegas, Jose Guillermo Rodriguez-Chong, Pablo D Treviño-Valdez, Oscar Tamez-Rivera

**Affiliations:** Pediatric Residency Program, Programa Multicéntrico de Especialidades Médicas ITESM- SSNL, Tecnológico de Monterrey. Escuela de Medicina y Ciencias de la Salud. Monterrey, México, Monterrey, Nuevo Leon, Mexico; Instituto Tecnólogico y de Estudios Superiores de Monterrey, Guadalajara, Jalisco, Mexico; ITESM, Guadalajara, Jalisco, Mexico; Tecnologico de Monterrey, Escuela de Medicina y Ciencias de la Salud, Monterrey, Nuevo Leon, Mexico; Tecnologico de Monterrey, Escuela de Medicina y Ciencias de la Salud, Monterrey, Nuevo Leon, Mexico

## Abstract

**Background:**

The growing threat of antimicrobial resistance is associated with the overprescription of broad-spectrum antibiotics. Antimicrobial stewardship programs (ASP) promote the responsible use of antibiotics. Their successful implementation requires adequate infrastructure and interdisciplinary efforts. ASPs in Mexico (Mx) are still scarce, particularly in pediatric centers.

**Table 1.** Diagnostics that justified use of Vancomycin

**Figure:**
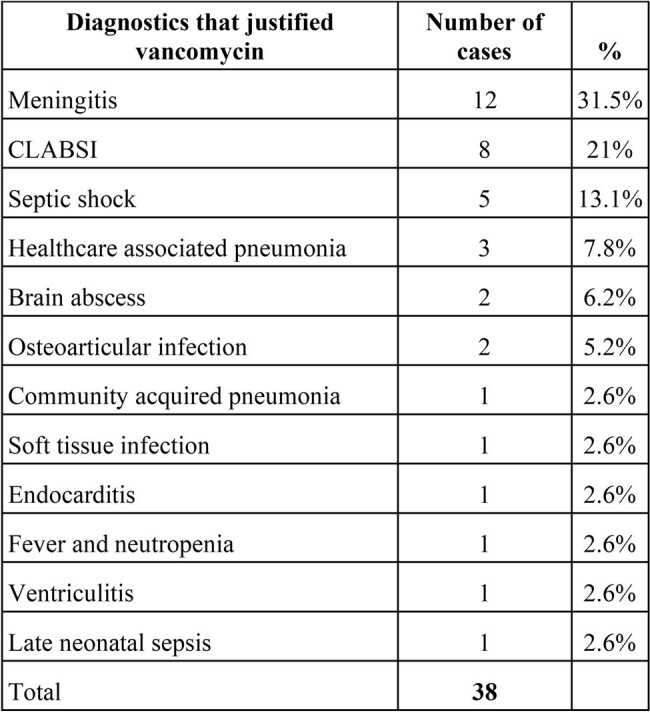

Total of diagnostics founded on 71 Stewardship Interventions

**Methods:**

Ambispective study to compare Vancomycin (VA) use before-after implementing a pilot 9-month handshake (HS) ASP at the pediatric reference hospital in NL, Mx. Stewardship interventions were applied from Jan to Oct 2022 among patients >1 mo and < 16 yr hospitalized with clinical suspicion/confirmation of Gram-positive infection, candidates to receive or currently receiving VA. PID specialists performed morning HS rounds. Prescription was labeled as correct/incorrect according to IDSA guidelines. Interventions were stratified as VA prevention and early discontinuation. VA use (DOT/1000 PD) from Jan to Oct 2021 was compared with t-Student test and ANOVA.

**Table 2.**
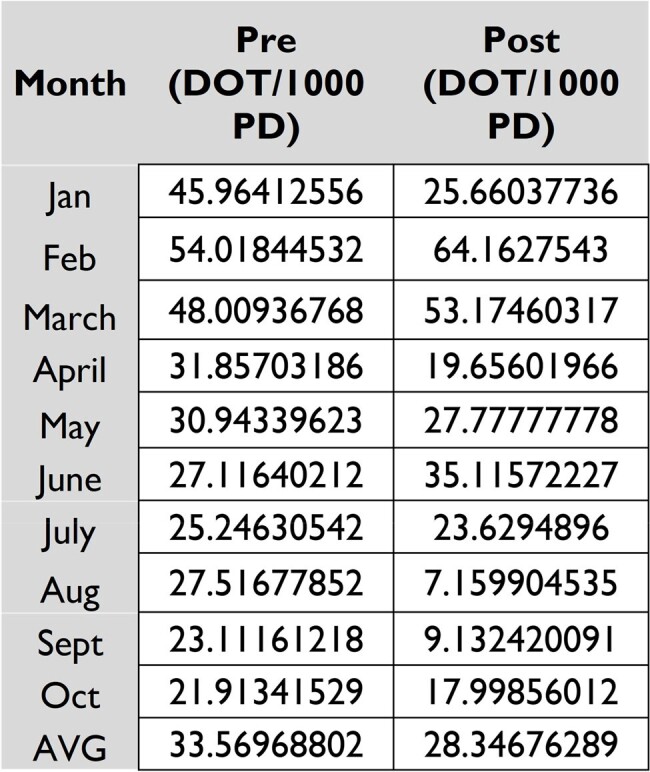
DOT/1000 PD Pre and Post the 9-month Pilot Hand-Shake Stewardship Program

Analysis of the DOT/1000 PD Compared of the Same Population at the Pediatric Reference Hospital in NL, Mx on a 9-month Period.

**Results:**

A total of 92 stewardship interventions were applied. Of these, the incorrect use of VA was prevented before its prescription in 21 cases. VA prescription was incorrect in 46% of the cases, in which early discontinuation was ordered. Correct use of VA (54%) occurred in patients with meningitis (31.5%), CLABSI (21%), septic shock (13%), among others (Table 1). SA was isolated in 14 patients. MRSA prevalence was 35.7% (5/14). Both prevention and early discontinuation of VA led to a decrease of 5.2 DOT/1000 PD (8.2 vs 3.6 DOT/1000 PD) (p=0.06) (Table 2).

**Conclusion:**

Implementing a HS ASP for 9 months in a pediatric reference hospital in Mx decreased the use of VA by 5.2 DOT/1000 PD. Although this was a short pilot program, implementing ASP reduced the use of broad-spectrum antibiotics. Extending the ASP duration may achieve lower VA use and statistical significance. The number of PID specialists in Mx is limited, which highlights the importance of ASP. Multidisciplinary efforts are required to reduce antimicrobial overuse.

**Disclosures:**

**All Authors**: No reported disclosures

